# Bioactive Alkaloids from Genus *Aspergillus*: Mechanistic Interpretation of Their Antimicrobial and Potential SARS-CoV-2 Inhibitory Activity Using Molecular Modelling

**DOI:** 10.3390/ijms22041866

**Published:** 2021-02-13

**Authors:** Fadia S. Youssef, Elham Alshammari, Mohamed L. Ashour

**Affiliations:** 1 Department of Pharmacognosy, Faculty of Pharmacy, Ain Shams University, Cairo 11566, Egypt; fadiayoussef@pharma.asu.edu.eg; 2 Department of Pharmacy Practice, College of Pharmacy, Princess Nourah bint Abdulrahman University, Riyadh 84428, Saudi Arabia; ejalshammari@pnu.edu.sa; 3 Department of Pharmaceutical Sciences, Pharmacy Program, Batterjee Medical College, Jeddah 21442, Saudi Arabia

**Keywords:** alkaloids, antimicrobial activity, *Aspergillus*, molecular modelling

## Abstract

Genus *Aspergillus* represents a widely spread genus of fungi that is highly popular for possessing potent medicinal potential comprising mainly antimicrobial, cytotoxic and antioxidant properties. They are highly attributed to its richness by alkaloids, terpenes, steroids and polyketons. This review aimed to comprehensively explore the diverse alkaloids isolated and identified from different species of genus *Aspergillus* that were found to be associated with different marine organisms regarding their chemistry and biology. Around 174 alkaloid metabolites were reported, 66 of which showed important biological activities with respect to the tested biological activities mainly comprising antiviral, antibacterial, antifungal, cytotoxic, antioxidant and antifouling activities. Besides, in silico studies on different microbial proteins comprising DNA-gyrase, topoisomerase IV, dihydrofolate reductase, transcriptional regulator TcaR (protein), and aminoglycoside nucleotidyl transferase were done for sixteen alkaloids that showed anti-infective potential for better mechanistic interpretation of their probable mode of action. The inhibitory potential of compounds vs. Angiotensin-Converting Enzyme 2 (ACE2) as an important therapeutic target combating COVID-19 infection and its complication was also examined using molecular docking. Fumigatoside E showed the best fitting within the active sites of all the examined proteins. Thus, *Aspergillus* species isolated from marine organisms could afford bioactive entities combating infectious diseases.

## 1. Introduction

Recently, marine-derived fungi have gained significant attention as promising therapeutic approaches for the treatment of a wide array of human ailments and as successful tools for drug discovery [1]. This is mainly attributed to their richness by a diverse array of secondary metabolites comprising terpenoids, alkaloids, peptides, lactones and steroids. These promising activities are represented by antiviral, antibacterial, anti-inflammatory and anticancer activity [2]. The significant diversity in physical and chemical structure of the environment where the marine-derived fungi grow has greatly reflected by the vast structural and functional variation in their produced secondary metabolites and their biological activities [3]. Meanwhile, marine-derived fungal metabolites displayed a promising physico-chemical behavior and oral-bioavailability, constituting a safer therapeutic alternative when compared to synthetic molecules that are considerably important in the process of pharmaceutical dosage form formulation [4,5]. Moreover, many alkaloids were previously isolated from marine fungi and showed a vast array of biological activities [6,7,8,9].

Genus *Aspegillus* represents a widely spread genus of fungi that are highly popular of possessing a potent medicinal potential comprising mainly antimicrobial, cytotoxic and antioxidant activities that are highly attributed to its richness by alkaloids, terpenes, steroid and polyketons. These secondary metabolites reflect the considerable importance of genus *Aspergillus* both in the scientific and pharmaceutical industries levels [10].

Thus, this review aimed to comprehensively explore the diverse alkaloids isolated and identified from different species of genus *Aspergillus* that were found to be associated with different marine organisms regarding their chemistry and biology. Classification was done on the basis of alphabetical arrangement of species. Around 174 alkaloid metabolites were reported, 66 of which showed important biological activities mainly comprising antiviral, antibacterial, antifungal, cytotoxic, antioxidant and antifouling activities. In addition, data illustrating the bioactive alkaloids obtained from previously mentioned fungal strains, their sources and biological properties are compiled in Table 1 for better representation of the collected data. A pie chart illustrating the different biological activities for the bioactive alkaloids of genus *Aspergillus* was also provided. Besides, in silico studies on different microbial proteins were done for sixteen alkaloids that showed anti-infective potential for better mechanistic interpretation of their probable mode of action. In addition, the inhibitory potential of these compounds vs. Angiotensin-Converting Enzyme 2 (ACE2) as an important therapeutic target combating COVID-19 infection and its complication was also examined using molecular docking to it can act as a guide for researchers who wish to continue exploring the anti-infectious potential of alkaloid derived from genus *Aspergillus*.

## 2. Diverse Alkaloids Isolated and Identified from Different Species of Genus *Aspergillus* and Their Biology in Alphabetical Arrangement of Species

### 2.1. A. carneus

**Figure 1:**
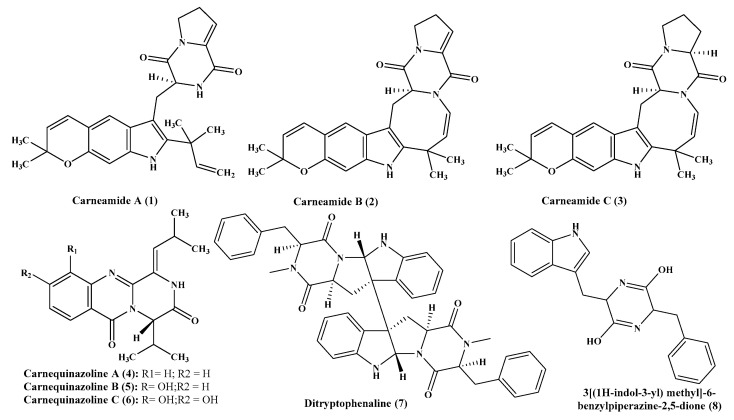
Alkaloids isolated from *Aspergillus carneus* and *A. flavus.*

*Aspergillus* species are highly popular due to the presence of a wide variety of alkaloids belonging to diverse classes. Prenylated indole and quinazolinone alkaloids were isolated from *A. carneus*, a marine associated *Aspergillus* species, while the former is represented by carneamides A-C (**1**–**3**); however, the latter is represented by carnequinazolines A-C (**4**–**6**). Unfortunately, none of the isolated compounds revealed any antimicrobial or cytotoxic activities (Figure 1) [11].

### 2.2. A. flavus

In depth phytochemical investigation of *A. flavus* resulted in the isolation of two alkaloids of diketopiperazine class; namely, ditryptophenaline (**7**) and 3[(1H-indol-3-yl) methyl]-6-benzylpiperazine-2,5-dione (**8**). Their structures were unambiguously elucidated via accurate analysis of their spectroscopic data (Figure 1) [12].

### 2.3. A. fumigatus

**Figure 2:**
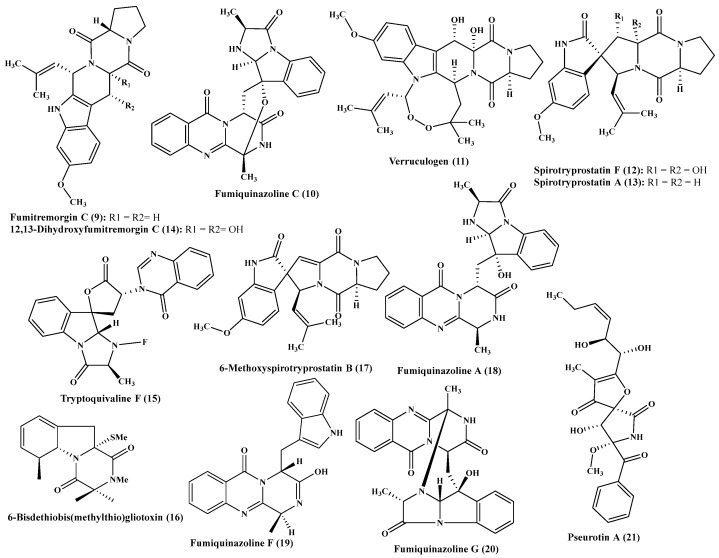
Alkaloids isolated from *Aspergillus fumigatus.*

**Figure 3:**
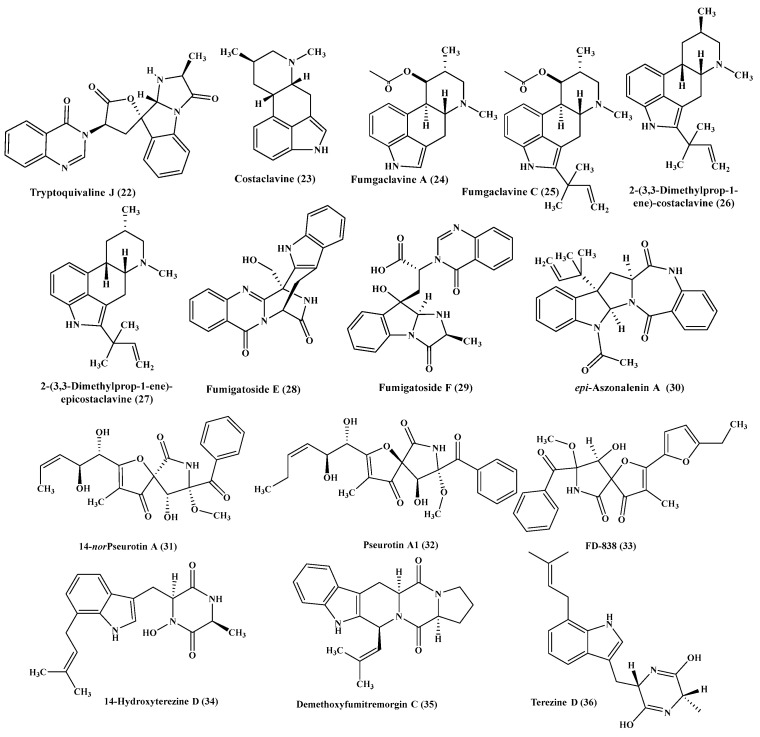
Alkaloids isolated from *Aspergillus fumigatus* (cont’d).

Regarding *A. fumigatus*, it revealed the presence of plethora of alkaloids (Figure 2 and Figure 3) which are represented by fumitremorgin C (**9**), fumiquinazoline C (**10**), verruculogen (**11**), spirotryprostatin F (**12**), spirotryprostatin A (**13**), 12,13-dihydroxyfumitremorgin C (**14**), tryptoquivaline F (**15**), 6-bisdethiobis(methylthio)gliotoxin (**16**), 6-methoxyspirotryprostatin B (**17**). Compounds (**9**–**13**) effectively stimulate the growth of buckwheat seedlings at very low concentration estimated by 10–16 μM [13], however compounds (**16**–**17**) potentiate the growth of seedling roots of *Zea mays* in an effective manner [14]. Besides, fumiquinazoline A (**18**), fumiquinazoline C (**10**), fumiquinazoline F (**19**), fumiquinazoline G (**20**), pseurotin A (**21**), as well as tryptoquivaline J (**22**), were isolated from the same *Aspergillus* species. All the isolated six alkaloid metabolites showed a substantial inhibitory activity on tsFT210 cells proliferation; however, fumiquinazoline C and pseurotin A effectively inhibited BEL-7402, A-549, P388 and HL60 proliferation [15]. Furthermore, costaclavine (**23**), fumgaclavine A (**24**) and C (**25**), which were isolated from *A. fumigatus* together with two new alkaloids of indole type 2-(3,3-dimethylprop-1-ene)-costaclavine (**26**) and 2-(3,3-dimethylprop-1-ene)- epicostaclavine (**27**) revealed mild cytotoxic effect vs. P388, mouse leukemia cancer cells [16]. Meanwhile, new alkaloids isolated from *A. fumigatus* displayed a potent antimicrobial activity in which fumigatoside E (**28**) showed antifungal activity vs. *Fusarium oxysporum* with MIC equals 1.56 µg/mL. Besides, fumigatoside F (**29**) showed a considerable activity against *A. baumanii* with MIC equals 6.25 µg/ mL. In addition, fumiquinazoline C (**10**), fumiquinazoline G (**20**) and *epi*-aszonalenin A (**30**) showed moderate potential against *A. baumanii,* two *S. aureus* strains, *K. pneumonia*, *Fusarium oxysporum cucumerinu* and *momordicae* with MIC ranging from 1.5 to 25 µg/mL, in which streptomycin was used as a positive antibacterial standard drug and nystatin as a positive antifungal standard drug. Meanwhile, fumiquinazoline G showed the greatest activity with MICs equal to 1.56 and 0.78 µg/mL, against the *Staphylococcus aureus* strains [17]. Moreover, another study carried on *A. fumigatus* revealed that some of its alkaloids showed a notable in-vitro antiproliferative effect such as pseurotin A (**41**), 14-norpseurotin A (**31**), pseurotin A1 (**32**), FD-838 (**33**) however other alkaloids as 14-hydroxyterezine D (**34**), demethoxyfumitremorgin C (**35**) and terezine D (**36**) were also isolated [18]. 

### 2.4. A. nidulans

**Figure 4:**
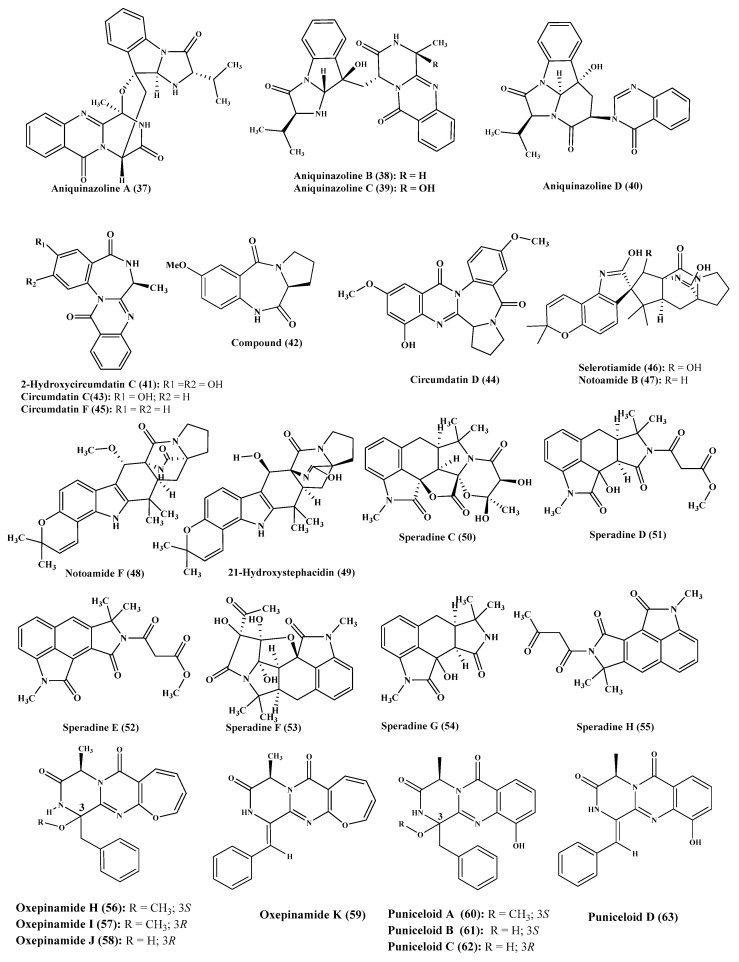
Alkaloids isolated from Aspergillus nidulans, A. ochraceus, A. oryzae and A. puniceus.

Furthermore, four new alkaloids of quinazolinone type which are aniquinazolines A-D (**37**–**40**) were isolated from *A. nidulans,* which was associated with the leaves of *Rhizophora stylosa,* marine plant. Compounds (**37**–**40**) revealed a significant lethal effect on brine shrimp displaying LD50 of 1.27, 2.11, 4.95 and 3.42 μΜ, in a respective manner which is superior to the positive control colchicine. However, none of these compounds exhibited any antibacterial activity vs. *Escherichia coli* and *S. aureus* or any cytotoxic effect on HL-60, BEL-7402, K562 and MDA-MB-231 cancer cells (Figure 4) [19].

### 2.5. A. ochraceus

*A. ochraceus* is also a rich source of alkaloids of benzodiazepine type such as 2-hydroxycircumdatin C (**41**) and 2,3-dihydro-7-methoxy-1Hpyrrolo[2,1-c][1,4] benzodiazepine -5,11(10H,11aH)-dione (**42**) that are considered to be new naturally occurring alkaloids in addition to known compounds as circumdatin C (**43**), circumdatin D (**44**), circumdatin F (**45**), selerotiamide (**46**) and notoamide B (**47**). 2-Hydroxycircumdatin C showed a potent antioxidant power as evidenced by its IC50 that is estimated by 9.9 μM in DPPH radical scavenging assay showing a superior activity comparable to butylated hydroxytoluene, a familiar synthetic positive control with IC_50_ = 88.2 μM. However, circumdatin C and D displayed mild antioxidant activity with IC_50_ value more than 100 μM in the same assay. None of compounds (**41**–**47**) revealed any antibacterial potential against *S. aureus* or *E. coli* or antifungal effect vs. *A. niger* [20]. Concerning *A. ostianus,* two heptacyclic alkaloidal compounds of stephacidin class were isolated from its culture medium namely notoamide F (**48**) and 21-hydroxystephacidin (**49**) (Figure 4) [21].

### 2.6. A. oryzae

Meanwhile, a series of oxindole alkaloids represented by speradines C-H (**50**–**55**) were isolated from *A. oryzae,* which showed a mild cytotoxic effect on HeLa cell line (Figure 4) [22,23].

### 2.7. A. puniceus

Furthermore, *A. puniceus* is a good source of new alkaloids from which eight new diketopiperazine-type alkaloids were isolated from the extract of its culture broth. Four of these new diketopiperazine alkaloids contain oxepin moiety however the other four contain quinazolinone moiety. The formers are represented by oxepinamides H-K (**56**–**59**); meanwhile, the latters were represented by puniceloids A-D (**60**–**63**) (Figure 4). Noteworthy to highlight that all the new eight isolated compounds revealed a potent transcriptional stimulation of liver X receptor α displaying EC_50_ ranging between 1.7 and 50 µM with puniceloids C and D showed the highest agonist behavior [24].

### 2.8. A. sulphureus

**Figure 5:**
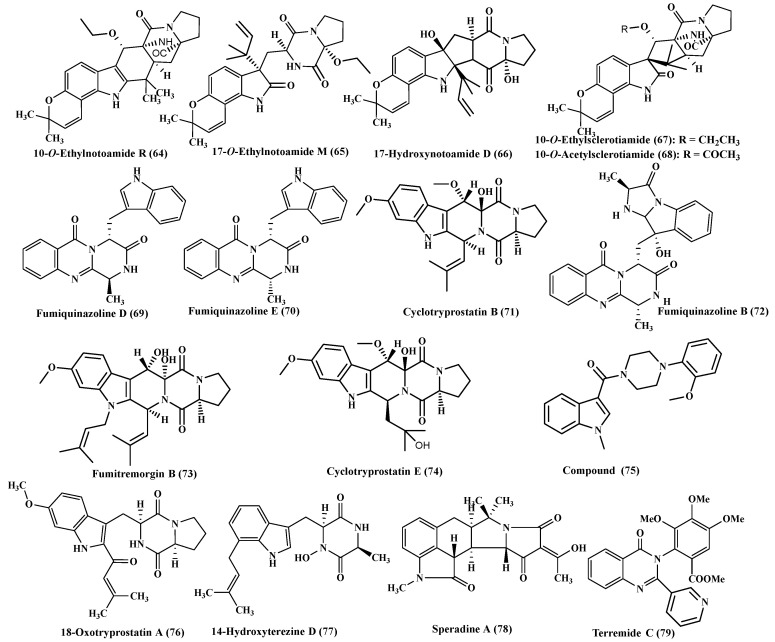
Alkaloids isolated from *A. sulphureus*, *A. sydowii*, *A. tamari* and *A. terreus*.

Additionally, the coculture of *A. sulphureus* and *Isaria feline* resulted in the isolation of five new alkaloids which are of prenylated indole class, 10-*O*-ethylnotoamide R (**64**), 17-*O*-ethylnotoamide M (**65**), 17-hydroxynotoamide D (**66**), 10-*O*-ethylsclerotiamide (**67**), and 10-*O*-acetylsclerotiamide (**68**) (Figure 5). It was found that 17-*O*-ethylnotoamide M effectively prohibits the colonization of 22Rv1, human prostate cancer cells, at a concentration of 10 µM which is considered as a non-toxic concentration [25].

### 2.9. A. sydowii

*A. sydowii* was subjected to an intense phytochemical investigation that led to the isolation of many indole alkaloids, fumiquinazoline D and E (**69**–**70**) and cyclotryprostatin B (**71**) in addition to 12,13- dihydroxyfumitremorgin C (**14**), fumiquinazoline A (**18**), fumiquinazoline F (**19**) and fumiquinazoline G (**20**) (Figure 5)**.** These compounds were tested for their antifouling activity via assessing their inhibitory effect on the settlement of *B. neritina* larvae, at a concentration of 25 μg/mL, fumiquinazoline D, fumiquinazoline G and cyclotryprostatins B showed significant antifouling activity [8].

Besides, fumiquinazoline B (**72**), fumiquinazoline C (**10**), fumitremorgin B (**73**), cyclotryprostatin E (**74**) and [4-(2-methoxyphenyl)-1-piperazinyl][(1methyl-1H-indol-3-yl)]- methanone (**75**) were also isolated from *A. sydowii.* Compound (**10**) revealed cytotoxic activity against P388, HL60, A549, FT210 and BEL-7402 with IC_50_ ranging between 1 × 10^−5^ and 1 × 10^−4^ mol/L-1 [26,27]. Additionally, 18-oxotryprostatin A (**96**), 6-methoxyspirotryprostatin B (**17**) and 14-hydroxyterezine D (**77**) were also isolated from *A. sydowii* in which 6-methoxyspirotryprostatin B showed mild cytotoxic activity against HL-60 cells displaying IC_50_ of 9.71 µM [28].

### 2.10. A. tamari and A. terreus

*A. tamari*, a marine derived fungal strain, also yielded a new alkaloid possessing oxindole pentacylcic skeleton termed speradine A (**78**) [29] meanwhile *A. terreus* culture extract afforded a new alkaloid which is terremide C (**79**) (Figure 5) [30].

### 2.11. A. versicolor

**Figure 6:**
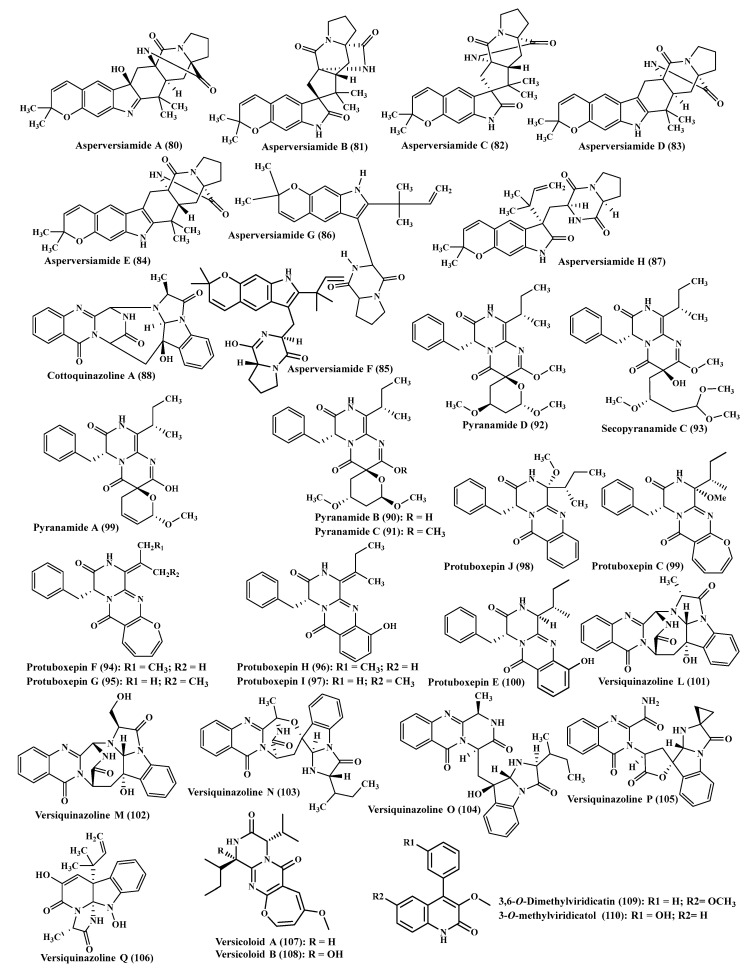
Alkaloids isolated from *A. versicolor*.

*A. versicolor* is highly popular by the presence of a large number of alkaloids (Figure 6), which are represented by asperversiamides A-H (**80**–**87**), which are indole alkaloids characterized by the presence of a linear fused prenyl groups and cottoquinazoline A (**88**) [7,31]. Asperversiamide G (**86**) displayed a significant anti-inflammatory potential evidenced by the pronounced inhibition of iNOS with IC_50_ value of 5.39 µM [7]. Additionally, ten new alkaloids of diketopiperazine class were isolated from *A. versicolor,* pyranamides A-D (**89**–**92**), secopyranamide C (**93**), protuboxepin F-J (**94**–**98**) in addition to previously isolated compounds which were protuboxepin C (**99**) and protuboxepin E (**100**). Protuboxepin G and E displayed mild cytotoxic activity vs. 786-O, OS-RC-2 and ACHN [9]. Further investigation of the coral derived fungus, *A. versicolor,* resulted in the exploration of six new alkaloids in the polycyclic form, which are versiquinazolines L-Q (**101**–**106**). Versiquinazolines P and Q displayed potent prohibition of thioredoxin reductase (TrxR) revealing IC_50_ of 13.6 and 12.2 µM, respectively being superior in activity relative to curcumin, the positive control, with IC_50_ of 25 µM accompanied by weak cytotoxic effect. This consequently, provides an evidence on the potential use of both compounds in the control of microenvironment of tumor progression and metastasis [32]. In addition, versicoloid A and B (**107**–**108**), 3,6-*O*-dimethylviridicatin (**109**) and 3-*O*-methylviridicatol (**110**) were also isolated from *A. versicolor,* in which versicoloid A and B displayed a potent anti-fungal activity with MIC of 1.6 µg/mL against *Colletotrichum acutatum* approaching cycloheximide, the positive control drug, that showed MIC of 6.4 µg/mL. [33].

### 2.12. A. westerdijkiae

**Figure 7:**
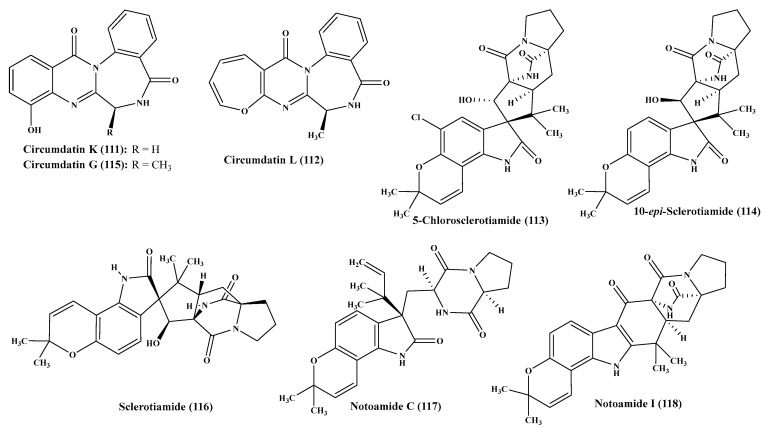
Alkaloids isolated from *A. westerdijkiae.*

Concerning *A. westerdijkiae,* circumdatins K and L (**111**, **112**), two new alkaloids of benzodiazepine type, 5-chlorosclerotiamide (**113**) and 10-*epi*-sclerotiamide (**114**), which are two new indole alkaloids carrying a prenyl moiety in addition to known alkaloid compounds which are circumdatin G (**115**), sclerotiamide (**116**), notoamide C (**117**), notoamide I (**118**) and circumdatin F (**45**) (Figure 7). However, none of the *A. westerdijkiae* isolated compounds showed cytotoxic effect vs. MCF-7, HL-60, A549 or K562 displaying IC_50_ greater than 10 μM. However, sclerotiamide showed a lethal effect on brine shrimps computed by 68% at 5 µg/mL [34].

### 2.13. Miscellaneous Aspergillus Species

**Figure 8:**
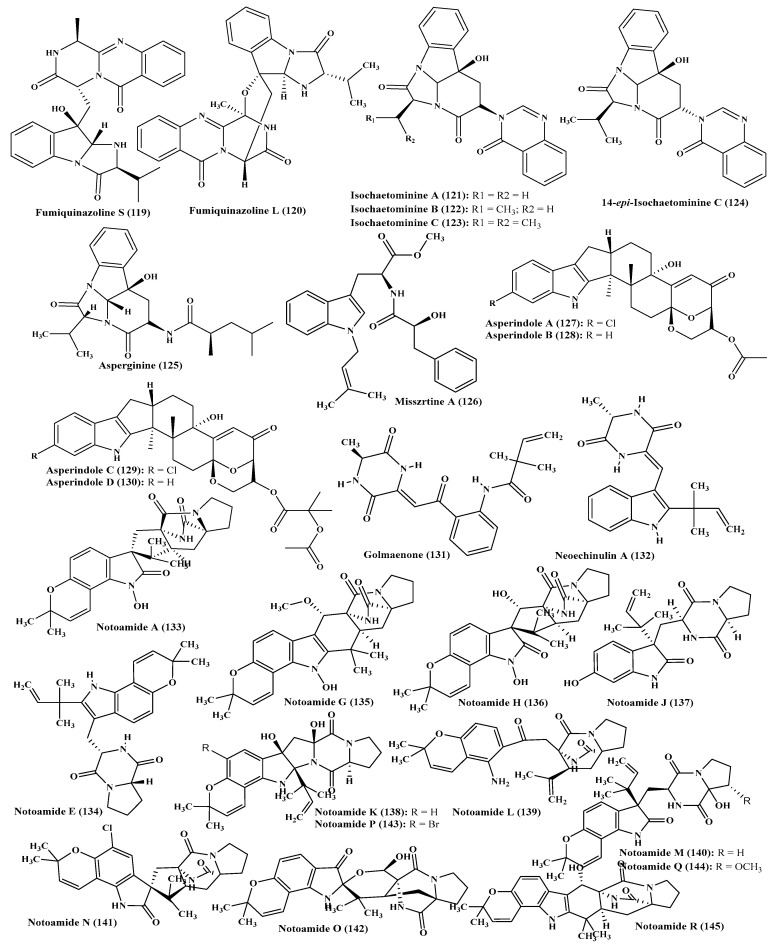
Alkaloids isolated from miscellaneous *Aspergillus* species.

Besides, a plethora of alkaloid compounds were isolated from miscellaneous *Aspergillus* species such as fumiquinazoline S (**119**), fumiquinazolines F (**29**) and L (**120**), isochaetominines A-C (**121**–**123**), 14-*epi*-isochaetominine C (**124**) (Figure 8). All these compounds revealed a mild inhibitory effect on Na(+)/K(+)-ATPase [35]. Additionally, asperginine (**125**), an alkaloid with a rare skeleton, and misszrtine A (**126**), an indole alkaloid with novel skeleton, possess phenylpropanoic amide arm attached to N-isopentenyl tryptophan methyl ester were isolated from two different *Aspergillus* species. The cytotoxic activity of the former was evaluated using MTT assay against human HCT116 and PC3 (prostate cancer cells) but it revealed no activity against the previously mentioned cell lines [36]. However the latter was assessed for its cytotoxic activity on HL60 and LNCaP and revealed a promising activity with IC_50_ value of 3.1 and 4.9 µM, respectively owing to the presence of indole nitrogen [37].

Besides, asperindoles A-D (**127**–**130**), new jndole alkaloids possessing diterpene structure, were also isolated from *Aspergillus* species. Asperindoles C and D possess a 2-hydroxyisobutyric acid moiety; however, asperindole A revealed a potent cytotoxic activity on both hormone therapy-resistant and sensitive PC-3 (Human prostate cancer cells) in addition to 22Rv1 cancer cells (human prostate carcinoma epithelial cell line) at low concentrations calculated in micromolar [38]. Golmaenone (**131**), new alkaloid with diketopiperazine skeleton, and neoechinulin A (**132**) were also isolated from *Aspergillus* species (Figure 8). Both compounds revealed a potent antioxidant activity evidenced by their IC_50_ values which are 20 and 24 µM, respectively in 1,1-diphenyl-2- picrylhydrazyl radical scavenging activity assay comparable to that of ascorbic acid (IC_50_ = 20 µM). Their antioxidant behaviour was further consolidated by their high UV-A (320–390 nm) protecting capability with ED_50_ values equal to 90 and 170 µM, respectively exceeding that of oxybenzone, the most popular consumed sunscreen (ED_50_ = 350 µM) [39]. Additionally, a series of prenylated indole alkaloids, notoamide A (**133**), notoamide B (**47**), notoamide C (**117**), notoamide E (**134**), notoamide F (**68**), notoamides G and H (**135**–**136**), notoamide I (**118**), notoamide J-R (**137**–**145**) were isolated from mussel-associated *Aspergillus* species. Notoamides A-C revealed a notable cytotoxic effect vs. cancer cells meanwhile notoamide I revealed a weak cytotoxic effect against HeLa cells with IC_50_ = 21 μg/mL. Additionally, notoamide A revealed a potent lethal effect on brine shrimps estimated by 63.0% at 5 µg/mL [40,41,42]. Additionally, versicolamide B and notoamides L–N were isolated from a marine derived *Aspergillus* species [41].

**Figure 9:**
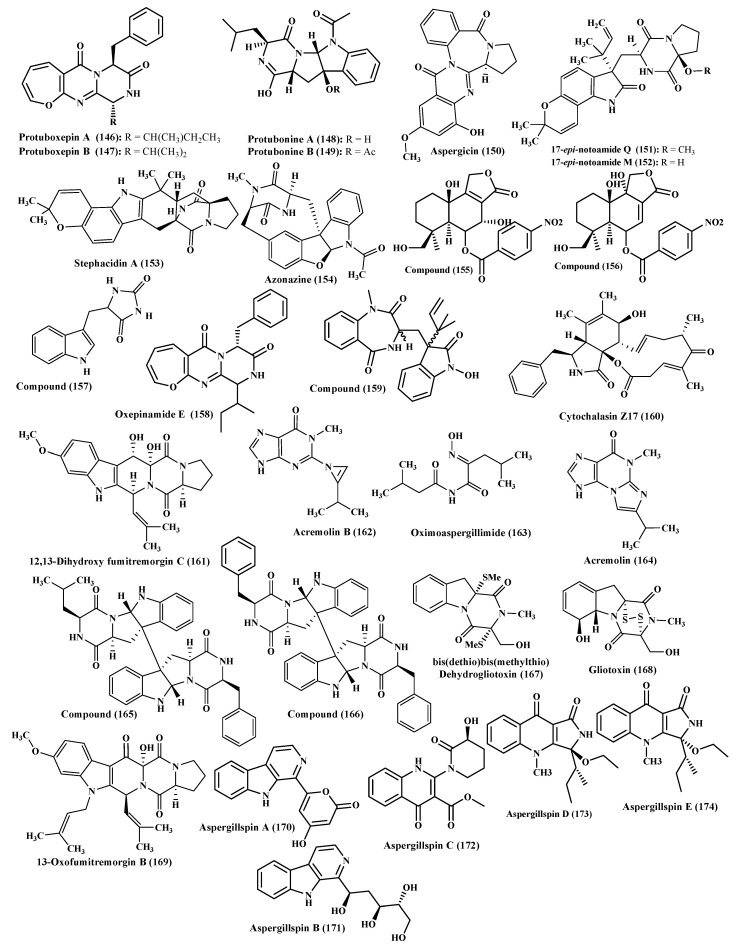
Alkaloids isolated from miscellaneous *Aspergillus* species.

Moreover, protuboxepins A and B (**146**–**147**) which possess oxepin moieties in addition to protubonines A and B, two diketopiperazine-type alkaloids (**148**–**149**) and aspergicin (**150**) were also isolated from *Aspergillus* species (Figure 9). Protubonines A exhibited a weak inhibition on cancer cells [43]; however, aspergicin showed a potent antibacterial activity against *Bacillus subtilis*, *Staphylococcus aureus* and *Staphylococcus epidermidis*, *Bacillus proteus*, *Bacillus dysenteriae* and *Escherichia coli* displaying MICs ranging from 15.62 to 62.50 μg/mL [44]. Additionally, two new prenylated indole alkaloids, 17-*epi*-notoamides Q and M (**151**–**152**) and stephacidin A (**153**) were also isolated from marine-associated *Aspergillus* species (Figure 9). None of them showed any cytotoxic activity vs. human promyelocytic leukemia HL-60 cell lines meanwhile only stephacidin A revealed antimicrobial vs. *Staphylococcus epidermidis* with MIC equals 14.5 μM [45]. Additionally, azonazine (**154**), 7α,14- dihydroxy-6β-p-nitrobenzoylconfertifolin (**155**), 9α,14-dihydroxy-6β-*p*- nitrobenzoylcinnamolide (**156**), 5-(1H-indol-3-ylmethyl) imidazolidine- 2,4-dione (**157**) and oxepinamide E (**158**) were also isolated from *Aspergillus* species. Compounds (**155**–**156**) displayed a potent inhibition to influenza virus strains H1N1 and H3N2, with IC_50_ of 36.0 and 12.0 μM, respectively, for compound (**155**) and 7.4 and 4.3 μM, respectively, for the two viruses for compound (**156**); meanwhile, compound (**154**) showed no activity [46,47]. Additionally, a new alkaloid, 3-((1-hydroxy-3-(2-methylbut-3-en-2-yl)- 2-oxoindolin-3yl)methyl)-1-methyl-3,4- dihydrobenzo[e][1,4] diazepine-2,5-dione (**159**) in addition to a known one, cytochalasin Z17 (**160**) were isolated from certain *Aspergillus* species. They revealed a potent antimicrobial activity vs. a number of microbes with compound (**159**) exerted a selective inhibition on *Vibrio harveyi*, *V. natriegens*, *V. proteolyticus*, *V. carchariae* showing MIC values between 0.0001 and 1 μg/mL; meanwhile, compound (**160**) showed a significant inhibition to *Roseobacter litoralis* showing MIC of 0.0001 μg/mL [48]. In addition, 12,13-dihydroxy fumitremorgin C (**161**), fumitremorgin C (**9**) and bis(dethio)bis(methylthio)gliotoxin (**16**) were isolated from certain *Aspergillus* species associated with the collected sediments existing in the northeast coast of Brazil (Figure 9) [49].

**Figure 10:**
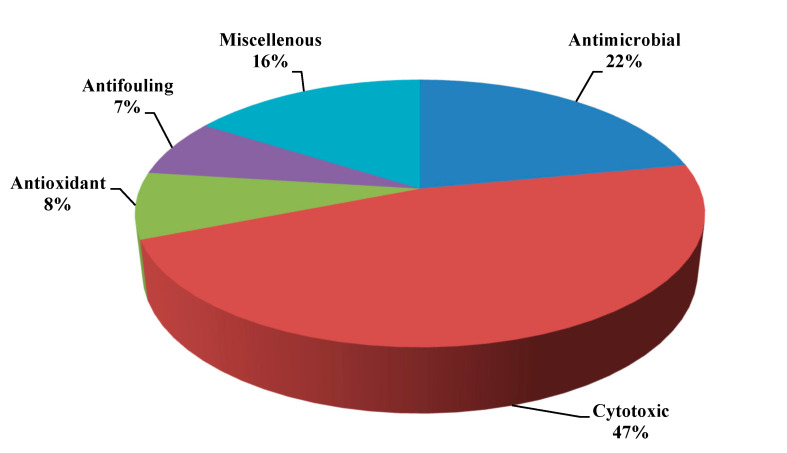
Percentages of different biological activities for the bioactive alkaloids of genus *Aspergillus.*

Additionally, new alkaloids, acremolin B (**162**), oximoaspergillimide (**163**) in addition to acremolin (**164**) were obtained as a result of the purification of the cultural extract of marine derived *Aspergillus* species; however, none of the compounds showed cytotoxic or antibacterial behavior [50,51]. Besides, new alkaloids, SF5280-415 (**165**), diketopiperazine dimer, and a closely related compound (**166**) were obtained from a marine associated *Aspergillus* species. Both compounds (**165**–**166**) revealed a potent inhibitory potential to protein tyrosine phosphatase 1B in an assay done using *p*-nitrophenyl phosphate as a substrate with IC_50_ values equal to 14.2 and 12.9 μM, for both compounds, respectively. Thus, both compounds can serve as natural candidates in the management of obesity as well as diabetes [52]. In an additional study carried on marine derived *Aspergillus* species, its isolated compounds bisdethiobis(methylthio)-dehydrogliotoxin (**167**), gliotoxin (**168**), 13-oxofumitremorgin B (**169**), fumitremorgin C (**9**), fumiquinazoline C (**10**), 12,13-dihydroxy-fumitremorgin C (**14**), bisdethiobis-(methylthio)gliotoxin (**16**), fumiquinazoline A (**18**), fumiquinazoline F (**19**), cyclotryprostatin B (**71**) and fumitremorgin B (**73**) were assessed for their anti-tuberculosis potential, cytotoxicity and antibacterial. Gliotoxin and 12,13-dihydroxy-fumitremorgin C displayed a considerable inhibition to *Mycobacterium tuberculosis* with MIC values less than 0.03 and 2.41 µM, respectively, In addition, gliotoxin exhibited potent cytotoxic activity vs. the three cell lines, A549, K562 and Huh-7 cell lines as evidenced by their IC_50_ values which are 0.015, 0.191 and 95.4 μM, respectively. Gliotoxin also revealed antibacterial potential vs. *Staphylococcus aureus*, *Escherichia coli* and *Salmonella* showing no antiviral or COX-2 inhibitory activity [53]. In another study performed on the marine gorgonian *Aspergillus*, aspergillspins A-B, new alkaloids with *β*-carboline moiety (**170**, **171**) and aspergillspins C-E (**172**–**174**), new alkaloids with quinolone structure were isolated and their antibacterial and cytotoxic activity were evaluated [54]. Besides, the hydroxypyrrolidine alkaloid preussin showed notable antibacterial activity [55] and diketopiperazine alkaloid mactanamide showed antifungal activity [56] were also isolated from genus *Aspergillus*. A pie chart illustrating the different biological activities for the bioactive alkaloids of genus *Aspergillus* was illustrated in Figure 10. Besides, prenylated indoles spirotryprostatins C–E and 13-oxoverruculogen and their cytotoxic effects upon a panel of cancer cell lines where spirotryprostatin E showed the most potent cytotoxic effect vs. MOLT-4, HL-60 and A-549 cells with IC50 ranging between 2.3–3.1μm [57].

**Table 1:** Diverse alkaloids isolated from marine derived fungal strains and their biological activities.

Compound	Genus	Biological Activity	References
Fumitremorgin C (**9**)	*A. fumigatus*	Notable antimicrobial activity against *Staphylococcus aureus*, methicillin-resistant *S. aureus*, and multidrug-resistant *S. aureus*	[58]
Fumiquinazoline C (**10**)	*A. fumigatus*	Inhibition of BEL-7402, A-549, P388 and HL60 proliferation	[15]
		Cytotoxic activity against P388, HL60, A549, FT210, BEL-7402	[15]
		Substantial activity against bacterial and fungal strains namely, *A. baumanii,* two *S. aureus* strains, *K. pneumonia*, *Fusarium oxysporum cucumerinu* and *momordicae*	[17]
12,13-Dihydroxy fumitremorgin C (**14**)	*A. fumigatus*	Potent inhibitory activity on *Mycobacterium tuberculosis*	[53]
		Notable antimicrobial activity against *Staphylococcus aureus*, methicillin-resistant *S. aureus*, and multidrug-resistant *S. aureus*	[58]
Fumiquinazoline G (**20**)	*A. fumigatus*	Substantial activity against bacterial and fungal strains namely, *A. baumanii,* two *S. aureus* strains, *K. pneumonia*, *Fusarium oxysporum cucumerinu* and *momordicae*	[17]
6- Bisdethiobis(methylthio) gliotoxin (**16**) 6-Methoxyspirotryprostatin B (**17**)	*A. fumigatus*	Potentiation of the growth of seedling roots of *Zea mays*	[14]
		Mild cytotoxic activity against HL-60 cells	[28]
Fumiquinazoline F (**19**)	*A. fumigatus*	Mild inhibitory effect on Na(+)/K(+) –ATPase	[35]
Pseurotin A (**21**)	*A. fumigatus*	Inhibition of BEL-7402, A-549, P388 and HL60 proliferation	[15]
Costaclavine (**23**)	*A. fumigatus*	Mild cytotoxic effect vs. P388	[16]
Fumgaclavine A (**24**)	*A. fumigatus*		
Fumgaclavine C (**25**)	*A. fumigatus*		
2-(3,3-Dimethylprop-1-ene)-costaclavine (**26**)	*A. fumigatus*	Mild cytotoxic effect vs. P388	[16]
2-(3,3-Dimethylprop-1-ene)-epicostaclavine (**27**)	*A. fumigatus*		
Fumigatoside E (**28**)	*A. fumigatus*	Significant antibacterial activity Antifungal potential against *Fusarium oxysporum*	[17]
Fumigatoside F (**29**)	*A. fumigatus*	Significant antimicrobial activity vs. *A. baumanii*	[17]
*epi*-Aszonalenin A (**30**)	*A. fumigatus*	Substantial activity against bacterial and fungal strains namely, *A. baumanii,* two *S. aureus* strains, *K. pneumonia*, *Fusarium oxysporum cucumerinu* and *momordicae*	[17]
Aniquinazolines A-D (**37**–**40**)	*A. nidulans*	Significant lethal effect on brine shrimp displaying LD50	[19]
2-Hydroxycircumdatin C (**41**)	*A. ochraceus*	Potent antioxidant power in DPPH radical scavenging assay	[20]
Circumdatin C (**43**)	*A. ochraceus*	Mild antioxidant power in DPPH radical scavenging assay	[20]
		Potent UV-A protective behavior	[16]
Circumdatin D (**44**)	*A. ochraceus*	Mild antioxidant power in DPPH radical scavenging assay	[20]
Speradines C-H (**50**–**55**)	*A. oryzae*	Mild cytotoxic effect on HeLa cell line	[22,23]
Puniceloids C and D (**62**–**63**)	*A. puniceus*	Potent transcriptional stimulation of liver X receptor	[24]
17-*O*-Ethylnotoamide M (**65**)	*Aspergillus*	Prohibition of the colonization of 22Rv1 (human prostate cancer cells)	[25]
Fumiquinazoline D and E (**69**–**70**)	*A. sydowii*	Significant antifouling activity by inhibiting the settlement of *B. neritina* larvae	[8]
Cyclotryprostatin B (**71**)	*A. sydowii*	Significant antifouling activity by inhibiting the settlement of *B. neritina* larvae	[8]
Asperversiamide G (**86**)	*A. versicolor*	Anti-inflammatory potential and pronounced inhibition of Inos	[7]
Protuboxepin G (**95**) and E (**100**)	*A. versicolor*	Mild cytotoxic activity vs. 786-O, OS-RC-2 and ACHN	[9]
Versiquinazolines P (**105**) and Q (**106**)	*A. versicolor*	Potent prohibition of thioredoxin reductase Weak cytotoxic effect	[32]
Versicoloid A and B (**107**–**108**)	*A. versicolor*	Potent anti-fungal activity vs. *Colletotrichum acutatum*	[33]
Circumdatin G (**115**)	*A. westerdijkiae*	Potent UV-A protective behavior	[16]
Sclerotiamide (**116**)	*A. westerdijkiae*	Pronounced lethal effect on brine shrimps	[34]
Fumiquinazolines S (**119**) and L (**120**)	*Aspergillus*	Mild inhibitory effect on Na(+)/K(+)-ATPase	[35]
Isochaetominines A-C (**121**–**123**)	*Aspergillus*		
14-*epi*-Isochaetominine C (**124**)	*Aspergillus*		
Misszrtine A (**126**)	*Aspergillus*	Promising cytotoxic activity on HL60 and LNCaP	[37]
Asperindole A (**127**)	*Aspergillus*	Potent cytotoxic activity on both hormone therapy-resistant and sensitive PC-3 as well as 22Rv1 cancer cells	[38]
Golmaenone (**131**)	*Aspergillus*	Potent antioxidant activity in 1,1-diphenyl-2-picrylhydrazyl (DPPH) radical scavenging activity assay	[39]
Neoechinulin A (**132**)	*Aspergillus*		
		High UV-A (320-390 nm) protecting capability	
		Notable inhibition on the barnacle larval settlement	[59]
		Potent cytotoxic effect on HeLa cells by inducing apoptosis	[60]
Notoamide A (**133**)	*Aspergillus*	Notable cytotoxic effect Potent lethal effect on brine shrimps	[40,41,42]
Notoamide B (**47**)	*Aspergillus*	Notable cytotoxic effect	
Notoamide C (**117**)	*Aspergillus*		
Aspergicin (**150**)	*Aspergillus*	Pronounced antimicrobial activity vs. *Bacillus subtilis*, *Staphylococcus aureus*, *Staphylococcus epidermidis*, *Bacillus proteus*, *Bacillus dysenteriae* and *Escherichia coli*	[44]
Stephacidin A (**153**)	*Aspergillus*	Selective antibacterial activity against *S. epidermidis*	[45]
7α,14- Dihydroxy-6β-p-nitrobenzoylconfertifolin (**155**)	*Aspergillus*	Effective inhibition on influenza virus strains H1N1 and H3N2	[46,47]
9α,14-Dihydroxy-6β-p-nitrobenzoylcinnamolide (**156**)	*Aspergillus*		
3-((1-hydroxy-3-(2-methylbut-3-en-2-yl)-2-oxoindolin-3yl)methyl)-1-methyl-3,4-dihydrobenzo[e][1,4]diazepine-2,5-dione (**159**)	*Aspergillus*	Promising antibacterial activity Selective inhibition on *Vibrio harveyi*, *V. natriegens*, *V. proteolyticus*, *V. carchariae*	[48]
Cytochalasin Z17(**160**)	*Aspergillus*	Promising antibacterial activity Potent inhibitory activity on *Roseobacter litoralis*	[48]
SF5280-415 (**165**)	*Aspergillus*	Potent inhibitory potential to protein tyrosine phosphatase 1B	[52]
Compound (**166**)	*Aspergillus*		
Gliotoxin (**168**)	*Aspergillus*	Potent inhibitory activity on *Mycobacterium tuberculosis* Potent cytotoxic activity vs. A549, K562 and Huh-7 Reasonable antibacterial activities against *Staphylococcus aureus*, *Escherichia coli* and *Salmonella*	[53]

## 3. Interpretation of the Antimicrobial Activity of Bioactive Alkaloids Using in Silico Studies

Many mechanisms explained the antimicrobial behavior of many anti-infective drugs such as prevention of nucleic acid, protein and cell wall synthesis, inhibition of functional cell membrane, as well as interfering with many metabolic processes [61,62,63]. Herein, molecular modelling was performed on six proteins which were downloaded from the protein data bank and are considered essential for growth, division, the survival of microbes and in the development of resistance using C-docker protocol [64,65,66]. These proteins are DNA-gyrase (PDB ID 4Z2D; 3.38 A°) from *Streptococcus pneumoniae*; topoisomerase IV (PDB ID 4Z3O; 3.44 A°) from *Streptococcus pneumoniae*; dihydrofolate reductase (PDB ID 4KM2; 1.4 A°) from *Mycobacterium tuberculosis;* β-lactamase (PDB ID 3NBL; 2.0 A°) from *Mycobacterium tuberculosis;* transcriptional regulator TcaR (protein) (PDB ID 4EJV; 2.9 A°) from *Staphylococcus epidermidis* and aminoglycoside nucleotidyl transferase (PDB ID 4WQL; 1.73 A°) from *Klebsiella pneumoniae.*

**Figure 11:**
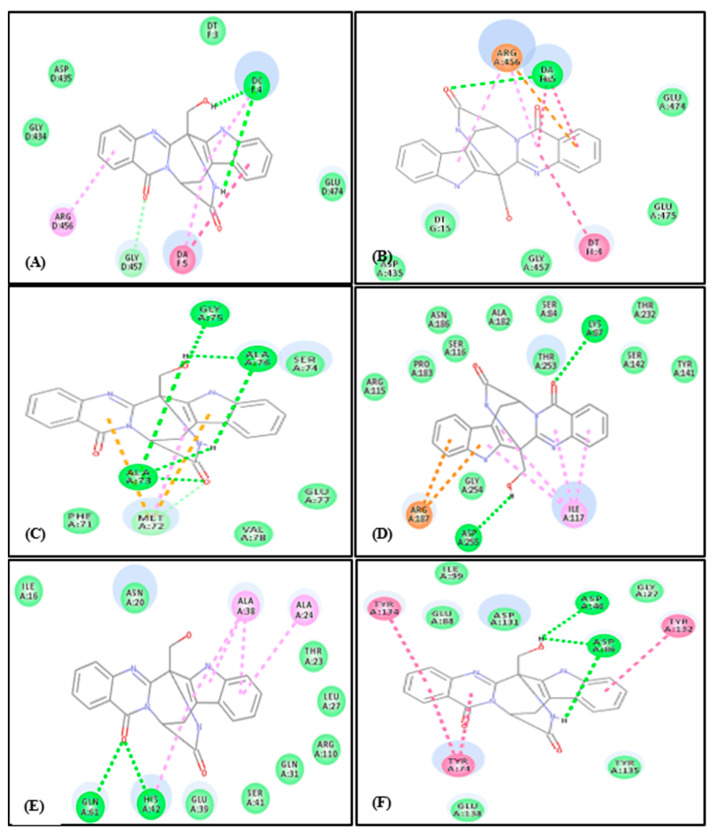
2D binding mode of Fumigatoside E (**28**) in the binding sites of DNA-gyrase (**A**), topoisomerase IV (**B**), dihydrofolate reductase (**C**), β-lactamase (**D**), transcriptional regulator TcaR (**E**) and aminoglycoside nucleotidyl transferase (**F**). Dotted green lines indicate H-bonds; dotted light green lines indicate C-H-bonds; dotted purple lines indicate π-bonds; dotted orange bonds indicate salt bridge formation.

Among all the examined compounds only fumigatoside E (28) showed the best fitting within the active sites of all examined proteins as evidenced by its free binding energies (∆G) that are equal to −14.18, −18.16, -5.02, −20.31, −10.84 and −17.59 Kcal/mol for DNA-gyrase, topoisomerase IV, dihydrofolate reductase, *β*-lactamase, transcriptional regulator TcaR and aminoglycoside nucleotidyl transferase, respectively. It showed in this aspect a superior activity comparable to levofloxacin and moxifloxacin, the potent DNA-gyrase, topoisomerase IV inhibitors, respectively with ∆G = −9.89 Kcal/mol for levofloxacin and −10.19 Kcal/mol for moxifloxacin, respectively whereas aspergicin (150) showed slight fitting. All of the other tested compounds showed unfavorable interaction within the active sites of the examined proteins manifested by the positive values of their free binding energies (∆G) (Table 2). The tight fitting of fumigatoside E can be interpreted by the virtue of formation of many tight bonds and interactions within the active sites (Figure 11).

Within the active site of DNA-gyrase, fumigatoside E formed two conventional H-bonds, a π–π bond, three π-alkyl bonds in addition to one C-H interaction and many Van der Waals interactions (Figure 11A). Regarding topoisomerase IV, fumigatoside E forms one conventional H-bond, three π–π bonds, two π-alkyl bonds in addition to many Van der Waals interactions and π-cation interaction with the amino acid residues at the active site (Figure 11B). Meanwhile, it forms five conventional H-bonds with Gly75, Ala76 and Ala73, two π- sulphur and one alkyl interactions with Met72 in addition to many Van der Waals interactions at dihydrofolate reductase active site (Figure 11C). Besides, fumigatoside E forms two H-bonds with Lys87 and Asp255, two π-cation interactions with Arg187 and four π-alkyl bonds with Ile117 at *β*-lactamase active site (Figure 11D). Concerning transcriptional regulator TcaR (protein), fumigatoside E forms two H-bonds with Gln 61 and His 42, and five π- alkyl bonds with Ala38, Ala24 and His42 and many Van der Waals interactions with the amino acid existing at the active site (Figure 11E). Three H-bonds with Asp46, Asp86 and four π–π interactions with Tyr74, Tyr132 and Tyr134 are formed between fumigatoside E and active site of aminoglycoside nucleotidyl transferase (Figure 11F). The notable binding of fumigatoside E with DNA-gyrase and topoisomerase active sites IV could greatly interpret its mode of antimicrobial via potent inhibition of both enzymes.

## 4. Probable SARS-CoV-2 Inhibitory Potential of Bioactive Antimicrobial Alkaloids Using in Silico Studies

COVID-19 infection relies upon host cell factors as Angiotensin-Converting Enzyme 2 (ACE2). The entrance of coronaviruses within the host cell is accomplished by the effective binding of the viral spike (S) proteins to cellular receptors that facilitate their cell entrance, viral attachment to the surface of target cells with subsequent infection triggering. SARS-S engages angiotensin-converting enzyme 2 (ACE2) as the entry receptor in which SARS-S/ACE2 interface was previously elucidated at the atomic level, and the effectiveness to bind with ACE2 was found to be a key determinant of SARS-CoV transmissibility. Thus the prohibition of ACE2 catalytic pocket by bioactive entities could alters the conformation of ACE2 in a manner that it could prohibit SARS-CoV-2 entrance within the host cells through ACE2 [67,68]. Thus, molecular modelling was performed for the sixteen alkaloids that previously displayed antimicrobial potential on Angiotensin-Converting Enzyme 2 (PDB ID 1R4L; 3.00 A°) which was downloaded from the protein data bank. Fumigatoside E (28) showed the most fitting within the active sites of ACE2 followed by aspergicin (150) displaying ∆G of −21.17 and −17.66 Kcal/mole, respectively (Table 3).

**Figure 12:**
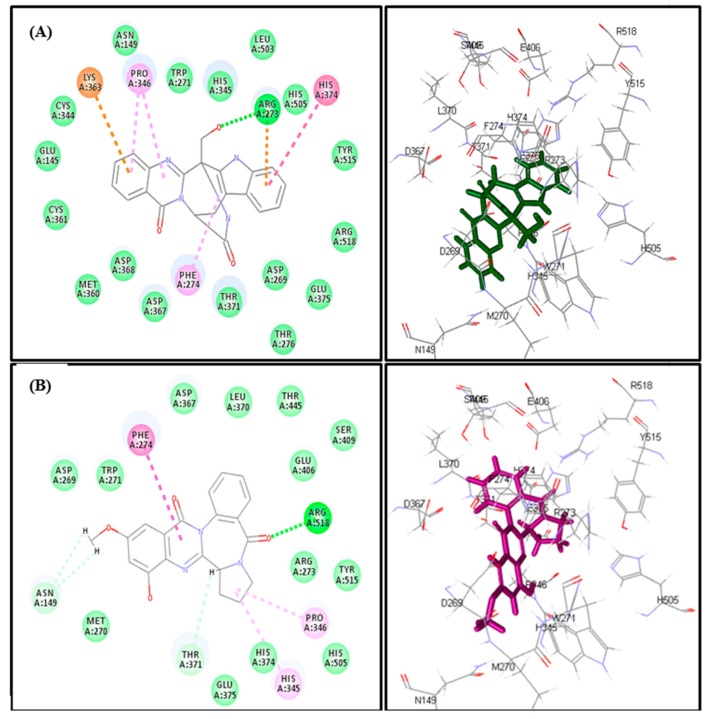
2D and 3D binding mode of Fumigatoside E (**28**) (**A**) and Aspergicin (**150**) (**B**) in the binding site ACE2.

Fumigatoside E (forms many tight interactions with the amino acid moieties at the active pocket of ACE2 represented by one H-bond with Arg273, π–π bond with His 379, three π-alkyl interactions with Pro346 and Phe274 in addition to the formation of two π-cation interactions with Lys363 and Ar273 (Figure 12A). Meanwhile, aspergicin forms one H-bond with Arg518, π–π bond with Phe 274, two π-alkyl interactions with His345 and Pro346, C-H bonds with Asn149 and Thr 371(Figure 12B).

## 5. Conclusions

Around 174 alkaloid metabolites were reported from genus *Aspergillus*, 66 of which showed important biological activities with respect to the tested biological activities mainly comprising antiviral, antibacterial, antifungal, cytotoxic, antioxidant and antifouling activities. Besides, in silico studies on different microbial proteins were done for sixteen alkaloids that showed anti-infective potential for better mechanistic interpretation for their probable mode of action. Fumigatoside E showed the best fitting within the active sites of all examined proteins as evidenced by its free binding energies. Additionally, fumigatoside E showed the most fitting within the active sites of ACE2 followed by aspergicin and thus could serve as bioactive candidates for combating SARS-CoV-2 infection. Further studies are to be conducted to examine the biological activities of the additional alkaloids that displayed no activity meanwhile in vitro followed by in vivo studies are to be performed to ascertain the results of molecular modelling.

## Figures and Tables

**Table 2:** Free binding energies (ΔG) in Kcal/mol of alkaloids isolated from *Aspergillus* and showed anti-infective potential using in silico studies on different microbial proteins.

Compound	DNA-Gyrase	Topoisomerase IV	Dihydrofolate Reductase	*β*-Lactamase	TcaR Protein	Aminoglycoside Nucleotidyl Transferase
Fumitremorgin C (**9**)	14.85	6.52	19.15	5.482	18.29	4.92
Fumiquinazoline C (**10**)	17.22	16.16	29.87	14.72	20.29	12.85
12,13-Dihydroxy fumitremorgin C (**14**)	14.50	5.80	27.40	5.52	15.85	7.34
Fumiquinazoline G (**20**)	25.62	15.66	37.59	21.60	24.82	17.97
Fumigatoside E (**28**)	−14.18	−18.16	−5.02	−20.31	−10.84	−17.59
Fumigatoside F (**29**)	0.39	−2.26	13.63	−11.27	6.69	−10.95
*epi*-Aszonalenin A (**30**)	27.73	29.20	43.62	29.47	32.30	21.33
Versicoloid A (**107**)	FD	FD	FD	FD	FD	FD
Versicoloid B (**108**)	FD	FD	FD	FD	FD	FD
Aspergicin (**150**)	−5.17	−6.94	3.11	−13.86	−2.93	−11.36
Stephacidin A (**153**)	FD	FD	FD	FD	FD	FD
Compound (**155**)	26.78	21.69	39.44	20.29	26.05	12.16
Compound (**156**)	29.18	25.50	44.72	14.35	30.68	26.91
Compound (**159**)	7.06	6.062	18.01	−4.81	6.38	−6.04
Cytochalasin Z17 (**160**)	46.79	45.14	63.26	43.15	50.64	39.36
Gliotoxin (**168**)	31.20	25.31	36.13	26.00	33.46	25.67
Levofloxacin	−9.89	ND	ND	ND	ND	ND
Moxifloxacin	ND	−10.19	ND	ND	ND	ND
Trimethoprim	ND	ND	−28.89	ND	ND	ND
Cefuroxime	ND	ND	ND	−61.80	ND	ND
Chloramphenicol	ND	ND	ND	ND	−29.02	ND
Kanamycin	ND	ND	ND	ND	ND	−73.94

ND: not done; FD: fail to dock.

**Table 3:** Free binding energies (ΔG) in Kcal/mole of alkaloids isolated from *Aspergillus* and showed anti-infective potential using in silico studies on Angiotensin-Converting Enzyme 2 (ACE2).

Compound	ΔG (Kcal/mole)
Fumitremorgin C (**9**)	−2.86
Fumiquinazoline C (**10**)	9.25
12,13-Dihydroxy fumitremorgin C (**14**)	−2.88
Fumiquinazoline G (**20**)	25.10
Fumigatoside E (**28**)	−21.17
Fumigatoside F (**29**)	−13.81
*epi*-Aszonalenin A (**30**)	24.62
Versicoloid A (**107**)	−1.86
Versicoloid B (**108**)	−2.66
Aspergicin (**150**)	−17.66
Stephacidin A (**153**)	−0.584
Compound (**155**)	18.13
Compound (**156**)	13.83
Compound (**159**)	−1.58
Cytochalasin Z17 (**160**)	43.80
Gliotoxin (**168**)	20.28

## Data Availability

Not applicable.
